# Giving patients a voice: implementing patient and public involvement to strengthen research in sub-Saharan Africa

**DOI:** 10.1136/jech-2019-212525

**Published:** 2020-01-31

**Authors:** Carol Bedwell, Tina Lavender

**Affiliations:** School of Health Sciences, University of Manchester, Manchester, UK

**Keywords:** maternal health, access to hlth care, health inequalities

Patient and public involvement (PPI) is recognised as a valuable tool in improving the quality and relevance of research.^1 2^ Defined as ‘research being carried out ‘with’ or ‘by’ members of the public rather than ‘to’, ‘about’ or ‘for’ them’,^2^ PPI is a method of involving patients and the public in the design, conduct and dissemination of research and services that affect them, providing for a more democratic approach and patient empowerment.^1 3^

Active involvement in research and healthcare is very much expected and is well established within many high-income settings. However, in low-income settings, PPI is in its infancy, with few researchers understanding the concept.^4^ This may lead to a failure to match health need with appropriate research,^5^ particularly when the research agenda is set by others, leading to acknowledged power imbalances.^6^ In such settings, empowerment of individuals is low and patients are not included in research design or conduct. In sub-Saharan Africa, gender inequalities mean that women have limited input into both healthcare-related decisions and research.^7^ Experience of working in these settings highlighted that this is the case even in research which is women-centred, such as maternity care.

In embarking on an National Institute for Health Research-funded programme of work related to prevention and management of stillbirth in sub-Saharan Africa, our research group recognised it was essential that women informed all stages of the research process. Stillbirth is stigmatised and seldom discussed in African culture, with women often blamed for the death of the baby, and is, therefore, considered a sensitive subject,^8^ which is challenging to research. In order for the research to meet its potential and to provide meaningful results, it was vital to gain women’s views to determine how such research could be best conducted. Thus, we introduced PPI from the start of the programme, with the aim of embedding PPI into the stillbirth programme of work in sub-Saharan Africa. Groups were established in each of the project’s partner countries; Kenya, Malawi, Tanzania, Uganda, Zambia and Zimbabwe, to reflect the varied cultures and contexts across the settings.

The majority of current guidance for conducting PPI relates to high-income settings, with no specific guidance available for developing PPI in low-income settings. After reviewing the available resources, we selected the Values and Principles Framework^9^ and Public Involvement Standards^10^ to guide our PPI strategy. The values incorporated in the framework, including respect, support, transparency, responsiveness, fairness of opportunity and accountability, were considered relevant across all cultures.^8^ The Public Involvement Standards relate to these values and are also largely applicable, incorporating inclusivity, working together, learning and communication. Project budgets, which included specific remuneration for time and expenses, were provided to participants, reflecting the value and recognition of their role.^11^ These were provided at agreed local rates in line with activities defined as research participation by INVOLVE.^11^

Incorporating PPI in a new setting involves overcoming social, cultural and practical challenges. A key challenge was the unfamiliarity of the concept to both women participants and researchers. Initial scoping work with the local research teams identified a lack of awareness of PPI and its potential benefits. Therefore, training was a key element in ensuring understanding of the aims of PPI for both researchers and group participants. In order to do this, we adopted the essential principles for training and support from the INVOLVE document, ‘Developing Training and Support for Public Involvement in Research’.^12^ We recognised that training needed to be adapted to the situation, and group participants required education sessions to explain the basics of research and their role as members of the public in providing input to support research. Researchers’ in-country had very limited understanding of PPI, and we provided specific training sessions and workshops led by an expert in PPI in low-income settings. Training and support have been ongoing during the project for both groups. INVOLVE^12^ acknowledges that support also includes emotional and psychological support, and the research team was mindful of this, given the sensitive nature of stillbirth. Initially, groups recognised the opportunity to support each other as a peer support group, and this allowed the groups to develop an identity and feel in a ‘safe’ environment to discuss stillbirth. On developing understanding of their role within the PPI group, the participants developed additional meetings, often after the formal PPI group, where they could debrief and discuss experiences. This is particularly relevant to these groups which are still childbearing and experiencing both live births and stillbirths during the progress of the study period. A strength of the cohesion of the groups is that members continue to attend regardless of the outcome of their own pregnancies. Culturally, patients, and particularly women, are rarely asked for their opinions in low-resource settings. One participant spoke on behalf of the PPI group in Tanzania.

We have not heard of PPI before, we are grateful for the opportunity to give our views as women and sincerely hope we can help develop research into stillbirth

One aspect in relation to PPI which service users in particular struggled with was the phrase ‘patient and public involvement’ and how it related to them, indicating that consideration needs to be given to how such terminology transfers to different cultures and whether more culturally relevant terms could be employed.

Successful recruitment of PPI group participants was ensured through a snowball sampling approach. The gold standard is to ensure hard-to-reach groups are represented,^13 14^ but this is a challenge where the concept of PPI is so unfamiliar and novel. We therefore did not specifically target groups traditionally viewed as hard to reach in the UK context.^14^ Hence, a pragmatic approach was taken to recruitment, bearing in mind that women in these settings are themselves a marginalised group.^15^ Managing expectations was important in establishing the PPI groups and was particularly relevant, given the newness of the concept in this setting.^12^ Ensuring clear group aims and roles was central to ensuring group focus and maintaining purpose.^12^ Providing the PPI groups with clear questions enabled researchers to gain meaningful input, for example, how to best access partners as participants in research. Group facilitators were vital in ensuring group cohesiveness, building relationships and maintaining contact with members between meetings. Facilitators were identified locally as individuals with skills to manage and maintain a group and were external to the research team in order to reduce, as far as possible, any power imbalances.^16^ This has been maintained as the groups have developed, with the groups having independence to determine meeting times and locations, along with extending invitations to members of the research team to attend and clarify points. Incorporating values of respect, support and fairness of opportunity has empowered participants to speak freely but without pressure to do so in a non-threatening and comfortable environment. Furthermore, in order to ensure the members feel comfortable, some groups are conducted in local language by the PPI facilitators who are bilingual. They then translate the overall responses into English for the UK team. Ensuring regular feedback to groups is acknowledged as crucial in ensuring that participants feel they are being heard and valued, providing motivation to continue to participate.^17^ During the data collection phase in Uganda, for example, the PPI facilitator highlighted the importance of this.

We cannot wait to hear the results; we want to make sure the research can really make a difference and we can tell people. We want to know the next steps.

This highlighted to the research team the importance of regular contact, which is maintained in many countries via What’s App group updates around the meetings.

PPI has been criticised for being tokenistic in that there is no real impact from participant involvement; rather it is a ‘tick box’ exercise.^13^ Poor reporting is also an issue^1^ and reporting checklists have been developed to address this issue.^18^ See table 1 for the GRIPP2 (Guidance for Reporting Involvement of Patients and the Public) checklist related to this project. For the purposes of this programme, impact was defined as a demonstrable contribution to research. This was measured as part of the monitoring and evaluation strategy, with indicators determined at the outset of the project. These included meeting agenda and minutes, records of feedback to and from groups, changes to the protocol design, as well as numbers of participants and groups and the frequency of group meetings. Evidence of impact includes changes to recruitment strategies in light of PPI feedback. For example, the PPI groups advised that culturally, women, particularly those in rural settings, could not make the decision to participate in research, and the partner and, frequently, the mother-in-law were required to be involved and give their agreement. Understanding these cultural issues enabled the research team to amend their recruitment strategies, taking time to visit women and families in the community to explain the study. The Uganda team were initially experiencing considerable difficulty in recruiting male participants to interview about experiences of stillbirth. Feedback from the PPI group enabled better understanding of how to access participants and recruitment rates increased, from less than one per week to three per week, ensuring the recruitment target was reached. Accessing traditional birth attendants (TBAs) was also an issue in Zambia and Tanzania. TBAs work in the communities, providing childbirth care for a fee. They are unqualified, work outside of the health systems and, despite a ban by Zambia in 2016, were continuing to provide care to women. Given the circumstances, identification of participants was a potential issue. The PPI group helped to identify TBAs and made initial contact, thus alleviating the potential concerns of the participants. They then introduced the researchers to individuals to discuss participation in the study. This enabled representation of the views of TBAs within the research, providing a pragmatic understanding of the current care provision around stillbirth. Additionally, PPI groups in all countries have reviewed and suggested amendments to topic guides where interview data were being collected. This included different ways of introducing the interview as many participants were concerned about confidentiality and anonymity. They also provided input into the questions asked, suggesting alternative wording, where translations of English words were inappropriate.

**Table 1:** GRIPP2 (Guidance for Reporting Involvement of Patients and the Public) short form checklist

Section and topic	Item	Reported on page number
1. Aim	To embed PPI into the stillbirth programme of work in sub-Saharan Africa	2
2. Methods	We recruited PPI participants through a snowball technique to groups in Kenya, Malawi, Tanzania, Uganda, Zambia and Zimbabwe. Training of both PPI group participants, PPI facilitators and researchers was required to clarify the purpose of PPI and the role of individuals within it. The groups needed to develop and skilled facilitators were key to this process.	2–4
3. Results	PPI involvement included the following contributions and impacts: Providing understanding of cultural issues and context in the study areas Advising on reaching potential participants Making initial contact and introducing researchers to potential hard-to-reach participants Providing advice on interview guides in terms of introducing the study and phrasing of questions Reviewing and commenting on analysed results Contributing to development of study interventions for the next phase of work A PPI participant was invited to sit on each in-country stakeholder group to provide PPI input. PPI groups held meetings in the local community to raise awareness of stillbirth.	5–7
4. Discussion and conclusions	The integration of PPI within this programme of work has provided impact in terms of a demonstrable contribution to the programme in terms of recruitment, data collection and design of interventions. In addition, PPI groups have raised awareness of stillbirth, both locally and nationally. The membership of the groups has provided individuals with a sense of worth and empowerment, enabling them to develop their own skills and contributions to research.	7
5. Reflections/critical perspective	PPI has been embedded into this ongoing programme of work. The groups are continuing to develop and provide additional input into the research process. Evaluation of the PPI groups and their contributions to the research process will be ongoing.	7

PPI, patient and public involvement.

The involvement of PPI groups has ensured research relevance and informed study recruitment, data collection and interpretation of results. The development of the research programme for the next phase has been substantially enhanced by the input from the now established PPI groups. This has led to design changes in determining the next phase of intervention development in the stillbirth programme. For example, the Tanzanian and Zambian PPI groups have provided the research team with confirmation that women would want to be asked about interventions, such as postmortem, to determine cause of death. Previous understanding in this setting was that this may be culturally unacceptable and upsetting, given the sensitive and hidden nature of stillbirth.

In addition to impact within the research programme, the PPI groups have also been influential in raising awareness of the issue of stillbirth in local communities and also at national level. The PPI group in Zambia contributed to the national stakeholder meeting, sharing their input into the project and also their own stories. This raising of awareness directly from the PPI participants led to the permanent secretary tabling a discussion of stillbirth in parliament. Giving women the opportunity to contribute at this level has the potential to have an impact on policy. At a local level, in-country PPI groups have been raising awareness in local communities and at church groups. These discussions are enabling a dialogue around stillbirth to develop, which will hopefully reduce some of the stigma related to this.

PPI participants have remained engaged throughout the process and have embraced the concept. For example, PPI representatives are, for the first time in this context, sitting on and contributing to stakeholder groups alongside health leaders. The fact that their voices are being heard is building confidence and empowering women participants. A participant from Tanzania commented:

Being able to discuss these matters with other women and knowing someone is finally listening is a relief. I feel I can speak out now and I want other women to feel the same.

In settings where women have little influence over health policy, women’s empowerment is recognised as vital in improving health for themselves and their families,^15^ while achieving equality in decision-making is a key principle of the sustainable development goals.^19^ Although this is an initial step into engaging women with experience of stillbirth in healthcare research and decision-making, many are already expressing a desire for greater involvement in setting research objectives. This is helping to move the power balance towards the groups and away from the researchers. The PPI groups are moving from a role of consultation to that of collaboration more quickly than we anticipated, with some groups, such as Kenya, expressing a desire to develop an advocacy group. Furthermore, individuals are developing collaboration roles with two PPI participants, from Malawi and Tanzania, currently involved as coapplicants on a further grant application, while others will be involved in supporting the research team in dissemination of research findings. Acknowledgement of participant views and active commitment to incorporating their recommendations will ensure a future research agenda more suited to women’s needs.^20^ Consequently, PPI in this context has had a twofold benefit: first, in informing the research, and second, in empowering under-represented groups to contribute to setting the future research agenda and developing appropriate healthcare strategies.

In summary, we are still learning the best way to embed PPI in low-income settings. Nevertheless, we have found that giving marginalised groups a voice in research is highly valuable, regardless of the setting. In order to ensure relevant and meaningful research with such populations, it is important that PPI is established as an integral part of research. It is vital that researchers working in low-income settings invest their time and commitment to include PPI when designing studies. It is also important that research funders provide adequate finance for PPI to be conducted effectively in low-income settings.

**Summary box**

- While patient and public involvement (PPI) in research is the gold standard in high-income settings, it remains alien in low-income settings, such as sub-Saharan Africa.
- Power imbalances in research are reinforced by the exclusion of PPI in the research process.
- We have successfully applied the INVOLVE principles in conducting PPI in research in sub-Saharan Africa.
- Social, cultural and practical challenges can be overcome to implement PPI in low-income settings.
- The benefits of PPI are amplified in these settings because of the traditional lack of consumer involvement.

## References

[1] Staniszewska S, Brett J, Mockford C, et al The GRIPP checklist: strengthening the quality of patient and public involvement reporting in research. Int J Technol Assess Health Care 2011;27:391–9. 10.1017/S0266462311000481 22004782

[2] Involve, 2018 Available: http://www.invo.org.uk [Accessed Jan 2019].

[3] Green G Power to the people: to what extent has public involvement in applied health research achieved this? Res Involv Engagem 2016;2:28 10.1186/s40900-016-0042-y 29507763 PMC5831888

[4] Luck A International public engagement. London: Wellcome Trust, 2016.

[5] Ditai J, Weeks A Patients’ roles in research: where is Africa? BMJ 2018;363:k4427 10.1136/bmj.k4427 30361390

[6] Nordling L Research: Africa's fight for equality. Nature 2015;521:24–5. 10.1038/521024a 25951267

[7] Asaolu IO, Alaofè H, Gunn JKL, et al Measuring women's Empowerment in sub-Saharan Africa: exploratory and confirmatory factor analyses of the demographic and health surveys. Front Psychol 2018;9:994 10.3389/fpsyg.2018.00994 29971030 PMC6018402

[8] Kiguli J, Munabi IG, Ssegujja E, et al Stillbirths in sub-Saharan Africa: unspoken grief. The Lancet 2016;387:e16–18. e13-e19 10.1016/S0140-6736(15)01171-X 26794074

[9] INVOLVE Public involvement in research: values and principles framework. Eastleigh: INVOLVE, 2015.

[10] Public Involvement Standards Development Partnership National standards for public involvement, 2018.

[11] INVOLVE Policy on payment of fees and expenses for members of the public actively involved with involve. Eastleigh: INVOLVE, 2016.

[12] INVOLVE Developing training and support for public involvement in research. Eastleigh: INVOLVE, 2012.

[13] Ocloo J, Matthews R From tokenism to empowerment: progressing patient and public involvement in healthcare improvement. BMJ Qual Saf 2016;25:626–32. 10.1136/bmjqs-2015-004839 PMC4975844 26993640

[14] INVOLVE Diversity and inclusion: What’s it about and why is it important for public involvement in research? Eastleigh: INVOLVE, 2012.

[15] WHO Addressing the Challenge of Women’s Health in Africa: Report of the Commission on Women’s Health in the African region. World Health Organisation, 2012.

[16] Ocloo JE, Fulop NJ Developing a 'critical' approach to patient and public involvement in patient safety in the NHS: learning lessons from other parts of the public sector? Health Expect 2012;15:424–32. 10.1111/j.1369-7625.2011.00695.x 21711471 PMC5060634

[17] Mathie E, Wythe H, Munday D, et al Reciprocal relationships and the importance of feedback in patient and public involvement: a mixed methods study. Health Expect 2018;21:899–908. 10.1111/hex.12684 29654644 PMC6186542

[18] Staniszewska S, Brett J, Simera I, et al GRIPP2 reporting checklists: tools to improve reporting of patient and public involvement in research. BMJ 2017;358:j3453 10.1136/bmj.j3453 28768629 PMC5539518

[19] United Nations Transforming our world: the 2030 agenda for sustainable development. New York: UN Publishing, 2015.

[20] Kennedy HP, Cheyney M, Dahlen HG, et al Asking different questions: a call to action for research to improve the quality of care for every woman, every child. Birth 2018;45:222–31. 10.1111/birt.12361 29926965

